# Pharmacological modulation of hyperarousal in insomnia associated with major depressive disorder: a narrative review of sedating antidepressants and orexin receptor antagonists

**DOI:** 10.3389/fpsyt.2026.1928430

**Published:** 2026-09-08

**Authors:** Yiwei Zhang, Hua Zhang, Jingkai Tang, Yibing Wang, Liang Wu

**Affiliations:** 1 Department of Rehabilitation Medicine, Peking University Shougang Hospital, Beijing, China; 2 Department of Sports and Health, Tianjin University of Sport, Tianjin, China

**Keywords:** dual orexin receptor antagonists, hyperarousal, insomnia, major depressive disorder, orexin receptor antagonists, personalized treatment, sedating antidepressants, sleep maintenance

## Abstract

Insomnia symptoms in major depressive disorder (MDD) are clinically heterogeneous, ranging from presleep rumination and prolonged sleep latency to repeated nocturnal awakenings and early-morning awakening. Their clinical significance and treatment implications may also differ between active MDD and residual insomnia symptoms during partial remission. This narrative review examines how these presentations may inform the use of sedating antidepressants and dual orexin receptor antagonists (DORAs). Sedating antidepressants, including mirtazapine, trazodone, doxepin, and other tricyclic antidepressants, differ in dose-dependent antidepressant activity, receptor profile, and adverse-effect burden. DORAs directly target orexin-mediated wake drive and have the strongest evidence for insomnia disorder, particularly sleep-maintenance outcomes, although evidence in active MDD remains limited. Because clinical presentations neither establish an underlying mechanism nor reliably predict treatment response, the proposed framework should be used as a hypothesis-generating aid rather than a validated treatment algorithm. Pharmacological selection should also consider suicide risk, substance-use history, comorbid sleep disorders, concomitant central nervous system depressants, and next-day functioning.

## Introduction

Population estimates vary substantially depending on whether insomnia is defined as symptoms or as a disorder. Symptom-based insomnia affects roughly one third of adults, whereas chronic insomnia disorder with daytime impairment is usually estimated at 6-10% (1–5). This distinction is clinically relevant. Reviews that combine transient sleep complaints with chronic insomnia disorder may overestimate some treatment needs while underestimating the burden associated with persistent insomnia symptoms and daytime impairment.

In MDD, sleep disturbance is part of the depressive syndrome but can also behave as a partially independent treatment target. Many depressed patients report insomnia symptoms, and population studies link chronic insomnia symptoms or insomnia disorder with later, recurrent, or persistent mood disorders (3, 6–9). This review focuses on adults with unipolar MDD and co-occurring insomnia symptoms or insomnia disorder, during either an active depressive episode or partial remission. Evidence from primary insomnia or broader insomnia-disorder populations is used only when it informs treatment positioning, and its indirectness is identified explicitly.

MDD-related insomnia symptoms are commonly described by timing, including sleep-onset difficulty, repeated awakenings, early-morning awakening, and non-restorative sleep. Although clinically useful, symptom timing does not identify the maintaining mechanism. Similar complaints may arise from depressive rumination, antidepressant activation, circadian misalignment, pain, or autonomic arousal. Clinical presentation should therefore be carefully distinguished from presumed pathophysiology.

The clinical significance of insomnia symptoms also extends beyond the night. Poor sleep can worsen concentration, irritability, fatigue, treatment adherence, and daily functioning. Residual insomnia symptoms and poor sleep quality are linked to impaired functioning and relapse vulnerability in depression (10–14). A treatment that improves sleep duration while worsening daytime cognition or alertness may therefore provide limited clinical benefit.

The bidirectional model remains useful, but it should not be treated as a uniform causal sequence. Depressive episodes can disturb sleep through rumination, anxiety, circadian disruption, neuroendocrine activation, and altered monoaminergic signaling. Persistent insomnia symptoms can then increase affective reactivity and reduce stress tolerance (15–18). In individual patients, however, insomnia symptoms may function as a precipitating factor, a residual symptom, or a consequence of earlier treatment decisions.

Pharmacological treatment is often considered because clinical care cannot always wait for ideal phenotyping. CBT-I may be unavailable, insomnia symptoms may be severe, or agitation may require more rapid symptom relief (19–23). Sedating antidepressants are attractive in this context because they may address mood and sleep simultaneously. Their use must be balanced against daytime sedation, cognitive dulling, weight gain, orthostatic symptoms, and fall risk (19, 24).

DORAs should not be interpreted as nonspecific sedatives or as first-line antidepressant augmentation. Their pharmacological rationale is narrower: by reducing orexin-mediated wake drive, they may target nocturnal alertness and sleep maintenance more directly than broad antihistaminergic or multi-receptor sedation. Trials in insomnia disorder support benefits for wake after sleep onset, total sleep time, and sleep efficiency (25–27). Evidence in active MDD (i.e., during an acute depressive episode) remains limited, particularly for suicidality, concomitant antidepressant use, substance-use history, and long-term mood outcomes.

Rather than comparing older and newer drug classes chronologically, this review links clinical sleep presentations, potential arousal mechanisms, and pharmacological considerations. It asks which clinical pattern, functional goal, and adverse-effect vulnerability might favor a particular strategy, rather than whether one drug class is globally preferable. This framework organizes the available evidence while preserving uncertainty about treatment prediction in patients with depression.

## Literature search and scope of the narrative review

This narrative review integrates evidence from sleep medicine, affective-disorder research, pharmacology, prescribing information, and clinical guidelines. It examines how insomnia symptoms in MDD may inform, but not determine, the selection of sedating antidepressants and DORAs. Targeted searches were conducted in PubMed/MEDLINE, Embase, PsycINFO, Web of Science Core Collection, the Cochrane Library, Google Scholar, and the reference lists of key reviews, meta-analyses, prescribing labels, and clinical guidelines. Evidence was selected for relevance to MDD-related insomnia, hyperarousal and sleep-wake regulation, sedating antidepressants, DORAs, clinical outcomes, and safety, and was classified by study design, population, dose, follow-up, outcome type, and major validity concerns. Full search strategies, eligibility criteria, screening procedures, study numbers, and the evidence-quality assessment framework are provided in **Supplementary Methods 1**.

## Insomnia symptoms in MDD as dysregulated hyperarousal

### Sleep-wake regulation and the depressive sleep phenotype

Sleep and wakefulness depend on the balance between sleep-promoting and wake-promoting systems. Sleep initiation partly requires sleep-promoting regions, including the ventrolateral preoptic area, to inhibit arousal circuits. Wakefulness is maintained by monoaminergic, cholinergic, histaminergic, and orexinergic pathways (2, 28–31). These pathways are not isolated switches. They interact with circadian timing, homeostatic sleep pressure, stress systems, emotional processing, reward circuitry, and cortical arousal (28, 30–32).

MDD overlaps with this network at several points. Serotonergic, noradrenergic, and dopaminergic pathways shape mood, motivation, reward, vigilance, and sleep architecture. Histaminergic neurons promote alertness. Orexin neurons stabilize wakefulness and integrate stress, reward, circadian, and metabolic signals (32, 33). Disturbance in any part of this network can influence both affective symptoms and sleep continuity. This is one reason antidepressants have uneven sleep effects: some reduce arousal and facilitate sleep, while others worsen insomnia or cause daytime somnolence (10, 17, 34, 35).

The clinical implication is straightforward but often overlooked: MDD-related insomnia symptoms should not be approached as one biological state. In this review, clinical phenotype denotes the observable pattern of symptom timing, presleep cognition, and daytime impairment. A biological phenotype would require objective evidence of a specific mechanism, such as neurophysiological, circadian, autonomic, or molecular markers. Sleep-onset difficulty, sleep-maintenance difficulty, and early-morning awakening can generate mechanistic hypotheses, but they cannot establish a biological phenotype (23, 36–39).

### Cognitive-emotional and physiological arousal are not interchangeable

The hyperarousal model is most useful when it is treated as a set of distinguishable processes rather than a single explanatory label. In MDD, the most clinically visible form is often cognitive-emotional arousal, including repetitive negative thinking, guilt, anticipatory worry, and difficulty disengaging from depressive content at bedtime. This framing is consistent with insomnia models that distinguish acute and chronic arousal processes (18, 29, 31, 40).

Physiological arousal requires a separate evidentiary basis. Sympathetic tone, heart rate variability, HPA-axis activity, and evening cortisol suggest stress-system activation in some patients, but findings are not uniform (17, 41, 42). The term arousal should therefore be specified carefully to avoid overstating the strength of evidence for a particular mechanism.

Cortical arousal and sleep perception add further complexity. Some patients show increased high-frequency EEG activity during sleep, whereas others show a discrepancy between subjective and objective sleep quality (38, 43). In MDD, negative appraisal may amplify this discrepancy. A treatment may improve objective sleep continuity without fully resolving subjective distress, or improve perceived sleep without normalizing sleep architecture.

In this review, hyperarousal is treated as a clinical and mechanistic framework rather than a diagnostic category. Relevant questions include whether wakefulness is maintained by repetitive cognition, physiological vigilance, orexin-mediated wake drive, or an activating medication regimen. These distinctions may guide pharmacological selection.

### Monoaminergic, histaminergic, and orexinergic pathways

Monoaminergic signaling links the depression and insomnia literatures, but it does not support a single classification of antidepressant sleep effects. Serotonin, norepinephrine, and dopamine shape mood, vigilance, motivation, and sleep architecture (10, 28). Some antidepressant effects that improve mood may worsen sleep early in treatment, whereas other receptor actions reduce arousal. Antidepressants should therefore be considered individually rather than as one sleep-related class.

Histamine provides a clear example of this trade-off. H1 receptor activation promotes wakefulness, whereas H1 antagonism produces sedation (19, 30). Mirtazapine and several tricyclic antidepressants can therefore help when sleep onset is delayed by distress or agitation. The same mechanism may be disadvantageous when appetite, weight, attention, falls, or morning functioning are major concerns.

Orexin provides a more specific entry point into wake stability. Orexin neurons project to monoaminergic and histaminergic arousal systems and help sustain wakefulness (32, 33). Blocking orexin receptors may be most relevant when the clinical presentation is repeated nocturnal awakening with persistent alertness. This rationale is plausible, but it remains insufficiently validated in depressed samples.

**Figure 1:**
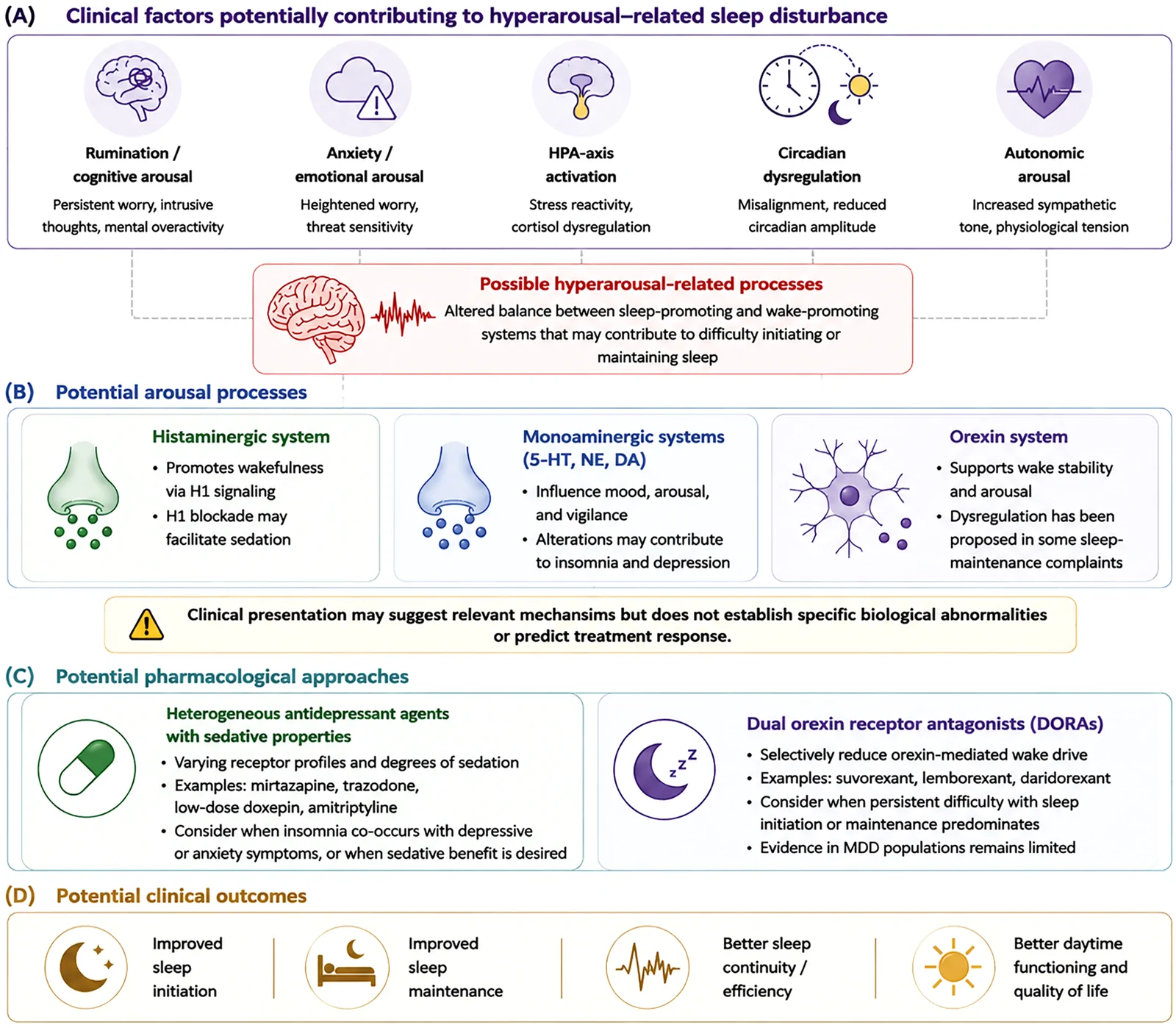
A hypothesis-generating framework for MDD-related insomnia. **(A)** Clinical factors potentially contributing to hyperarousal-related sleep disturbance; **(B)** potential arousal processes; **(C)** potential pharmacological approaches; and **(D)** potential clinical outcomes.

The clinical implication is that patients with the same insomnia label may require different treatment logic. One patient may primarily need reduction of depressive rumination before sleep, whereas another may require reduction of nocturnal wake drive. Treating these presentations as equivalent endpoints may obscure clinically meaningful differences in mechanism and treatment response. These relationships between clinical factors, potential arousal processes, and pharmacological approaches are summarized in **Figure 1**.

### Why the model matters for psychiatric outcomes

Sleep improvement and psychiatric recovery are related but not equivalent outcomes. A drug may increase sleep duration while causing next-day sedation, cognitive slowing, or emotional blunting. Another drug may improve sleep continuity without adequately addressing rumination, anxiety, or depressive hopelessness. In MDD, clinically meaningful sleep improvement should support energy, concentration, mood regulation, interpersonal functioning, and relapse prevention.

Research should test whether baseline clinical features modify treatment response. Relevant candidates include presleep rumination, wake after sleep onset, objective sleep fragmentation, and daytime sleepiness. This approach may be more informative than comparing mean changes in insomnia severity alone.

## Sedating antidepressants in depression-related insomnia symptoms

### Pharmacological rationale and best-fit use

Sedating antidepressants remain common because they appear to solve two problems at once: depressive symptoms and insomnia symptoms (19, 44). This is attractive when insomnia symptoms occur with rumination, anxious distress, appetite disturbance, agitation, and difficulty initiating sleep. These agents are pharmacologically diverse, however. Their sleep effects reflect different combinations of histaminergic, serotonergic, adrenergic, and cholinergic actions.

Mirtazapine is often associated with rapid subjective sleep improvement, partly through H1 antagonism and 5-HT2 receptor antagonism. Trazodone is frequently used off-label for insomnia and may improve sleep continuity through serotonergic and adrenergic mechanisms (44–47). Low-dose doxepin has a more selective H1 antagonist profile at hypnotic doses. Higher-dose tricyclic antidepressants, including amitriptyline and related agents, have broader anticholinergic and cardiovascular effects. Their sedative effects can be useful, but their adverse-effect burden can be substantial, especially in older adults or medically complex patients (21, 24).

This clinical positioning requires a dose-specific interpretation of sedating antidepressants. The same agent may have different implications when used at antidepressant doses than when used at low hypnotic or sleep-adjunct doses. Mirtazapine, for example, may be considered when depressive symptoms, anxious distress, appetite loss, and insomnia are part of the same acute presentation. By contrast, low-dose trazodone is better described as an adjunctive sleep-promoting strategy rather than as antidepressant treatment, and its use for chronic insomnia disorder should remain cautious in light of the American Academy of Sleep Medicine (AASM) guideline position. Low-dose doxepin has a narrower hypnotic role, with its strongest rationale in sleep-maintenance insomnia through H1 antagonism. These distinctions are important because they prevent sedating antidepressants from being treated as a single interchangeable category and align the framework with insomnia guidelines.

Within the hyperarousal framework, these drugs are broad arousal-reducing agents. They are most coherent when the aim is to reduce presleep distress, shorten sleep latency, and treat depressive-anxious symptoms at the same time. They are less coherent when the patient is already fatigued, cognitively impaired, fall-prone, metabolically vulnerable, or mainly troubled by repeated nocturnal awakening rather than difficulty falling asleep (19, 34, 44).

### What sleep improvement means in this drug class

Sedating antidepressants can reduce sleep latency and improve subjective sleep quality, but the strength of evidence differs by population and drug. Depression-focused data are most relevant for mirtazapine and related antidepressant studies (44, 46), whereas trazodone evidence is broader and mainly concerns sleep disturbance rather than MDD-specific outcomes (45, 47). Early subjective sleep improvement can still matter clinically. It may reduce distress, improve adherence, and make daytime routines more manageable.

Pharmacodynamic effects on sleep architecture are agent- and dose-dependent. Histamine H1 and 5-HT2 antagonism may improve sleep continuity, whereas serotonergic, noradrenergic, and anticholinergic actions can alter rapid eye movement sleep, slow-wave sleep, or nocturnal fragmentation. Sedation therefore cannot be assumed to restore physiological sleep architecture, and the implications for emotional processing and relapse remain uncertain (28, 34, 45, 46).

It is also difficult to separate sleep effects from antidepressant effects. If a patient sleeps better after starting mirtazapine, the improvement may reflect sedation, anxiolysis, appetite normalization, mood improvement, reduced arousal, or several of these mechanisms at once. This complexity is clinically useful when insomnia symptoms are embedded in a broader affective-arousal syndrome. It becomes a liability when the patient mainly needs targeted sleep maintenance without added daytime burden.

### Safety and functional trade-offs

The same receptor actions that promote sleep can impair next-day functioning. Daytime sedation, psychomotor slowing, reduced alertness, poor concentration, and fatigue are especially relevant in MDD because these symptoms overlap with the depressive burden (34). A treatment that improves nocturnal sleep but worsens daytime function may not meet the patient’s therapeutic goals.

Metabolic and cardiovascular effects require equal attention. Mirtazapine may increase appetite and weight. This may be helpful in a patient with appetite loss but may be undesirable in a patient with obesity, diabetes, or weight-related distress. Tricyclic antidepressants can cause anticholinergic effects, orthostatic hypotension, cardiac conduction concerns, and toxicity in overdose. Trazodone is often viewed as safer than older sedatives, but it can still cause next-day sedation, dizziness, orthostatic hypotension, and rare serious adverse events (19, 34, 44).

The clinical implication is not to avoid sedating antidepressants, but to use them selectively. They are reasonable when sleep-onset difficulty, anxiety, appetite loss, and depressive symptoms form one clinical picture. They are less attractive when residual sedation, cognitive impairment, fall risk, metabolic vulnerability, or a primary sleep-maintenance complaint dominates the case (19, 34, 45).

## Orexin receptor antagonists in MDD-related insomnia symptoms

### A different route to sleep, not a general antidepressant strategy

DORAs follow a different pharmacological logic. They target the orexin system, a central stabilizer of wakefulness (32, 33). Orexin neurons maintain alertness by activating noradrenergic, serotonergic, dopaminergic, cholinergic, and histaminergic pathways. Blocking orexin receptors reduces wake drive and allows sleep when homeostatic and circadian conditions are favorable. This differs from generalized sedation or GABAergic inhibition.

The distinction is clinically important in MDD. Many depressed patients are tired but not sleepy in a restorative sense. They may fall asleep, then wake repeatedly and remain alert for long periods. Others wake early and cannot return to sleep. These presentations may raise the clinical hypothesis of persistent wake drive and sleep-state instability. They do not demonstrate excessive orexin activity at the individual-patient level. DORAs may fit this hypothesis more closely than broad sedating antidepressants when sleep maintenance is the main residual complaint and presleep rumination is not dominant, but this reasoning remains inferential rather than biomarker validated (17, 33, 48).

Orexin signaling also interacts with stress responsiveness, reward processing, and emotional regulation, but evidence for effects beyond sleep remains provisional. Most DORA trials enrolled patients with insomnia disorder rather than active MDD, often underrepresenting unstable psychiatric illness, high suicide risk, complex polypharmacy, and concurrent antidepressant changes. Their sleep endpoints therefore cannot establish effects on depressive remission, emotional regulation, suicidality, or relapse. DORAs should be presented as sleep-focused agents with possible psychiatric relevance, not as antidepressants (17, 32, 48, 49).

### Sleep maintenance, sleep architecture, and daytime function

Randomized trials and meta-analyses in insomnia disorder show that suvorexant, lemborexant, and daridorexant improve sleep outcomes compared with placebo (25–27, 50–54). The most consistent benefits involve wake after sleep onset, total sleep time, and sleep efficiency. Sleep latency can also improve with some agents and doses, but the strongest rationale remains reduction of pathological wakefulness.

Sleep architecture is central to the appeal of DORAs. Because these agents reduce wake drive without broad central nervous system suppression, they may preserve more physiological sleep architecture than some traditional sedative strategies (55, 56). Studies of daridorexant and lemborexant suggest better sleep continuity without major disruption of rapid eye movement or slow-wave sleep. Whether this translates into better emotional regulation in MDD remains unanswered.

For depressed patients, better sleep maintenance may be valuable even when mood symptoms require separate treatment. Fragmented sleep worsens fatigue, concentration, irritability, and perceived non-restoration. A drug that reduces awakenings without excessive next-day sedation may be useful for residual insomnia symptoms during partial remission or ongoing antidepressant therapy. This role is especially relevant when the current antidepressant is otherwise effective or when sedating antidepressants have been poorly tolerated.

### Psychiatric safety and evidence gaps

Selectivity should not be equated with absence of clinical risk. Although DORAs avoid broad multi-receptor sedation, they remain hypnotic agents with potential next-day impairment, complex sleep behaviors, and abuse-related concerns. A history of substance use should therefore prompt careful assessment of misuse risk, concomitant use of alcohol or sedatives, dose escalation, and access to medication, rather than an automatic preference for DORAs. Clinicians should also consider driving or occupational demands, fall risk, comorbid sleep disorders, respiratory vulnerability, and concomitant sedating medications, particularly in older adults or patients receiving complex psychiatric regimens (25, 27, 57–59).

MDD-specific safety deserves explicit monitoring. FDA labeling for suvorexant and lemborexant warns about worsening depression and suicidal ideation, and depressed patients may have fluctuating suicide risk during medication changes (58, 59). Available clinical data do not establish DORAs as mood-destabilizing agents, but trials in general insomnia often underrepresent unstable psychiatric illness, high suicide risk, and complex polypharmacy (48, 49). In patients with active MDD, DORA use should therefore be preceded by assessment of current suicidal ideation, recent self-harm, bipolar-spectrum symptoms, rapidly worsening depression, substance-use risk, medication interactions, next-day impairment, and access to lethal means, with close follow-up after initiation or dose changes.

The most important evidence gap is not whether DORAs work for insomnia disorder. It is whether their benefits and risks translate to patients with active MDD and clinically significant insomnia symptoms. Future trials should include depressive symptom scales, anxiety measures, suicidality monitoring, relapse follow-up, daytime functioning, and pragmatic comparisons against sedating antidepressants, CBT-I, antidepressant optimization, or combination strategies (20, 26, 27, 48, 49, 60). The principal mechanistic and clinical differences between sedating antidepressants and DORAs are summarized in **Table 1**. The evidence base for these pharmacological and comparator strategies is summarized in **Supplementary Table 1**.

**Table 1:** Mechanistic and clinical comparison of sedating antidepressants and dual orexin receptor antagonists in MDD-related insomnia symptoms.

Domain	Sedating antidepressants	Dual orexin receptor antagonists (DORAs)
Core concept	Agent-specific sedating and antidepressant effects through multi-receptor actions.	Selective reduction of orexin-mediated wake drive.
Main targets	H1, 5-HT2A/2C, alpha1-adrenergic, serotonergic, and agent-specific anticholinergic effects; dose determines intent.	Dual antagonism of OX1R and OX2R.
Possible clinical signal	Sleep-onset difficulty with rumination, anxiety, agitation, appetite loss, or acute depressive distress.	Sleep-maintenance complaint with repeated awakenings, prolonged WASO, early awakening, or nocturnal alertness.
Potential advantage	May address mood, anxiety, appetite, and sleep when these targets coexist.	May improve sleep continuity with less broad receptor sedation than many sedating antidepressants.
Main concern	Daytime sedation, cognitive slowing, metabolic effects, orthostasis, anticholinergic burden, overdose toxicity, and heterogeneous evidence.	Limited active-MDD data; Schedule IV status; next-day impairment; label warnings about worsening depression and suicidal ideation.
Hypothesis-generating positioning	Consider when antidepressant-dose treatment is clinically indicated or when low-dose hypnotic use is guideline-concordant for a defined sleep target.	Consider for selected sleep-maintenance symptoms when mood treatment is otherwise addressed and safety screening is favorable.
Evidence gap	Few phenotype-stratified trials; low hypnotic doses and antidepressant doses are often conflated.	Few MDD-specific trials with suicidality, relapse, substance-use, polypharmacy, and antidepressant co-treatment outcomes.

DORAs, dual orexin receptor antagonists; H1, histamine H1 receptor; 5-HT, serotonin; MDD, major depressive disorder; OX1R, orexin-1 receptor; OX2R, orexin-2 receptor; TCAs, tricyclic antidepressants; WASO, wake after sleep onset.

## A phenotype-informed framework for treatment selection

### Start with the presenting sleep problem, then test the mechanism

Phenotype-informed treatment begins with the presenting sleep problem. Assessment should cover symptom timing, presleep cognition, daytime impairment, medication burden, comorbid sleep disorders, substance use, and patient goals. CBT-I remains a core treatment across phenotypes in the presence of insomnia disorder, conditioned arousal, maladaptive sleep beliefs, or irregular sleep behaviors. Medication may be added according to depressive phase, symptom urgency, access to CBT-I, and safety, but it should not displace behavioral treatment without a clinical reason (21–23, 29, 61, 62).

A cognitive-emotional hyperarousal presentation includes racing thoughts, guilt, anticipatory anxiety, and difficulty disengaging from depressive content. Sedating antidepressants may be considered when these symptoms coexist with an indication for antidepressant treatment, whereas CBT-I remains relevant when conditioned arousal and maladaptive beliefs maintain insomnia symptoms (17, 19, 29).

A nocturnal wakefulness presentation includes repeated awakenings, prolonged wakefulness, or early awakening with persistent alertness. A DORA may be considered when sleep maintenance remains the main target during partial remission or after antidepressant optimization. These features suggest, but do not establish, persistent wake drive, and active MDD still requires mood-focused care and safety monitoring.

Patient vulnerability can override phenotype matching. Sedating antidepressants require particular caution with daytime sleepiness, cognitive impairment, falls, metabolic risk, cardiovascular disease, polypharmacy, or overdose risk. DORAs require caution with substance-use risk, concomitant central nervous system depressants, respiratory vulnerability, complex sleep behaviors, and driving or occupational demands. Current suicidality, recent self-harm, bipolar-spectrum features, or rapidly worsening depression require psychiatric stabilization and close monitoring before either strategy (19, 25, 27, 34, 58, 59).

### Positioning sedating antidepressants and DORAs without false symmetry

Sedating antidepressants and DORAs are complementary rather than interchangeable. The former may serve both mood and sleep goals, whereas the latter primarily target sleep and wakefulness. Selection should therefore follow treatment phase, dose intent, symptom target, and safety profile (19, 44, 48).

**Figure 2:**
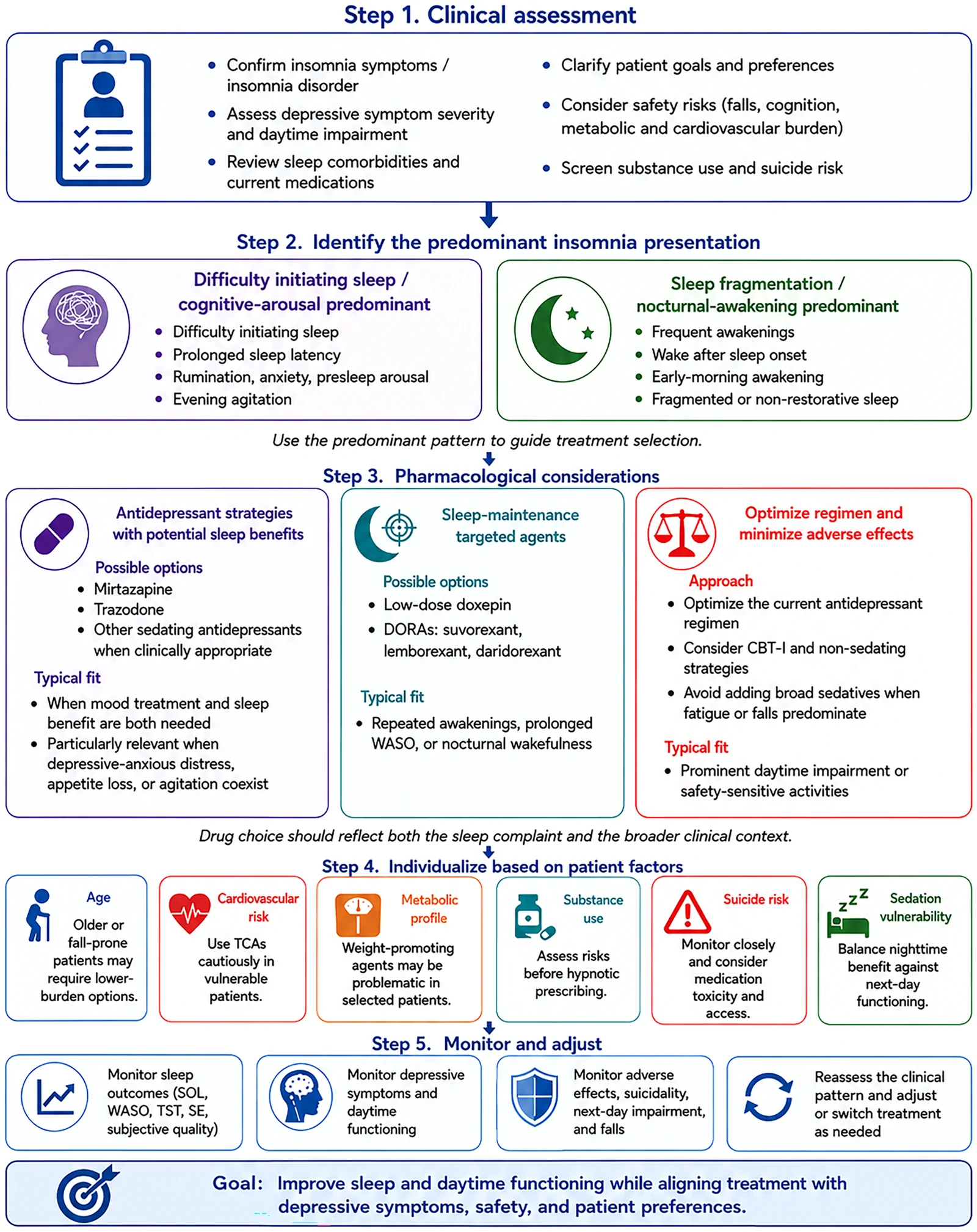
Clinical reasoning framework for the pharmacological treatment of insomnia symptoms in MDD. MDD, major depressive disorder; CBT-I, cognitive behavioral therapy for insomnia; DORA, dual orexin receptor antagonist; TCA, tricyclic antidepressant; SOL, sleep-onset latency; WASO, wake after sleep onset; TST, total sleep time; SE, sleep efficiency.

During active MDD, an antidepressant-dose sedating antidepressant may be rational when anxiety, appetite loss, agitation, and sleep-onset difficulty align with the broader treatment goal. During partial remission, persistent sleep-maintenance symptoms may instead prompt antidepressant optimization, CBT-I, low-dose doxepin, or a DORA. The proposed clinical reasoning framework is summarized in **Figure 2**.

### Practical scenarios

A patient with active MDD, marked anxiety, reduced appetite, and prolonged sleep latency may benefit from a sedating antidepressant when the same agent is appropriate for depression. Here, insomnia symptoms are part of a broader affective-arousal presentation, although daytime sedation and metabolic effects require monitoring (19, 44, 63).

A patient in partial remission who remains troubled by repeated awakenings may require a different approach. When presleep rumination is limited, CBT-I, evaluation for comorbid sleep disorders, or a DORA may better match the residual sleep-maintenance target, although DORA evidence remains largely extrapolated from insomnia disorder trials (26, 48).

When prolonged sleep latency, nocturnal awakenings, sleep-related worry, and irregular sleep habits coexist, medication alone is unlikely to be sufficient. CBT-I, circadian stabilization, antidepressant optimization, and short-term pharmacological support may need to be combined. The framework is intended to structure clinical assessment rather than replace clinical judgment (20, 29, 60). The phenotype-informed positioning of the principal pharmacological options is summarized in **Table 2**.

**Table 2:** Phenotype-informed positioning of pharmacological options in MDD-related insomnia symptoms.

Clinical presentation	Clinical hypothesis to consider	Possible clinical consideration	Main caution
Long sleep latency with rumination, anxiety, guilt, or presleep cognitive activity	Cognitive-emotional hyperarousal may be prominent, but symptoms do not prove mechanism	CBT-I is relevant; an antidepressant-dose sedating antidepressant may be considered when mood or anxiety treatment is also needed.	Residual sedation, cognitive slowing, appetite increase, and impaired daytime function.
Sleep-onset insomnia with appetite loss, agitation, or weight loss during an acute depressive episode	Diffuse affective and physiological arousal	Mirtazapine or another sedating antidepressant may fit selected patients when antidepressant treatment is indicated.	Metabolic effects and daytime sedation require monitoring.
Repeated awakenings, prolonged WASO, fragmented sleep, or early awakening with alertness	Persistent wake drive is a hypothesis, not a demonstrated biomarker	DORA, CBT-I, or antidepressant optimization may be considered when sleep maintenance is the main target.	Active MDD evidence remains limited; mood and suicidality must be monitored.
Primary sleep-maintenance insomnia without need for antidepressant-dose change	Sleep continuity rather than mood initiation may be the main target	Low-dose doxepin is guideline-concordant for sleep-maintenance insomnia; DORA may be considered in selected cases.	Do not conflate low-dose doxepin with antidepressant-dose TCAs.
Low-dose trazodone used mainly for sleep	Sleep-promoting adjunct rather than simultaneous antidepressant treatment	Use only after considering guideline disagreement, comorbidities, orthostasis, and next-day impairment.	AASM recommends against trazodone for chronic insomnia disorder.
Residual insomnia after mood response or partial remission	Sleep disturbance may be partially independent of active depressive syndrome	CBT-I, low-dose doxepin, DORA, dose-timing adjustment, or medication review may be considered.	Avoid assuming residual insomnia equals active MDD or orexin excess.
Sedation-vulnerable patient with fatigue, cognitive slowing, falls, driving risk, or polypharmacy	Daytime vulnerability may dominate the risk-benefit balance	Prioritize non-sedating strategies, CBT-I, medication simplification, and cautious dosing if medication is used.	Nocturnal improvement is insufficient if 24-hour function worsens.
Substance-use history or current controlled-substance risk	Abuse and misuse risk must be assessed explicitly	CBT-I and medication review are central; DORAs require caution because they are Schedule IV controlled substances.	Do not present DORAs as preferred solely because of substance-use history.
Current suicidal ideation, recent self-harm, bipolar-spectrum features, or rapidly worsening depression	Psychiatric safety question overrides phenotype matching	Perform suicide-risk assessment, stabilize mood risk, limit access to lethal medication quantities, and monitor closely before any hypnotic strategy.	FDA labels for suvorexant and lemborexant warn about worsening depression and suicidal ideation.

CBT-I, cognitive behavioral therapy for insomnia; DORA, dual orexin receptor antagonist; MDD, major depressive disorder; TCA, tricyclic antidepressant; WASO, wake after sleep onset.

## Discussion

This review frames insomnia symptoms in MDD as heterogeneous clinical presentations that may reflect distinct arousal processes. Its contribution is to organize treatment selection around symptom timing, daytime impairment, dose intent, and adverse-effect vulnerability rather than a single hierarchy of sedative potency.

The available evidence addresses related but distinct populations and outcomes. Epidemiological and polysomnographic studies link insomnia with mood disorders (8, 18). Guidelines support CBT-I and selected medications for insomnia disorder (21–23, 39, 60, 64), while DORA trials demonstrate sleep benefits in that population (25–27, 50–53). However, the applicability of these findings to patients with active MDD remains partly inferential.

Sedating antidepressants and DORAs should therefore not be ranked along a single continuum. Sedating antidepressants may address depressive-anxious distress, sleep latency, and appetite but can worsen fatigue, cognition, metabolic risk, or falls. DORAs more selectively reduce wake drive and may suit sleep-maintenance symptoms, yet most trials do not address suicidality, polypharmacy, emotional instability, or relapse in active MDD.

The most clinically useful distinctions are between sleepiness and fatigue, and between active MDD and residual insomnia during partial remission. These distinctions support targeted assessment without implying that symptoms identify a mechanism.

## Limitations and future directions

Direct comparisons among sedating antidepressants, low-dose doxepin, DORAs, CBT-I, and antidepressant optimization in well-characterized active MDD are lacking (20, 48, 49, 54, 62). Evidence also varies across doses, comparators, follow-up, concomitant medications, and sleep measures. Most DORA trials concern insomnia disorder, while psychiatric subgroup analyses cannot isolate effects on mood, suicidality, emotional regulation, or relapse from sleep improvement (25, 34, 44, 45, 48, 49).

Future trials should stratify participants by sleep-onset difficulty, sleep-maintenance difficulty, early-morning awakening, rumination, anxiety, daytime sleepiness, cognitive function, and treatment phase. Outcomes should include mood remission, relapse, suicidality, daytime function, cognition, quality of life, adherence, and patient preference, rather than only nocturnal sleep measures (20, 22, 23, 54, 61, 64).

Insomnia symptoms during active MDD should not be assumed to represent the same clinical entity as chronic insomnia disorder or residual symptoms during partial remission. Until direct comparative trials address these distinctions, the framework can support cautious clinical reasoning but not a validated prescribing sequence (11, 16, 65).
