# Supplementary material for: Pharmacological modulation of hyperarousal in insomnia associated with major depressive disorder: a narrative review of sedating antidepressants and orexin receptor antagonists
Source: Front Psychiatry. 2026 Sep 8;17:1928430. doi: 10.3389/fpsyt.2026.1928430 (PMC13595994; doi:10.3389/fpsyt.2026.1928430)
Supplement: Supplementary file 1 [file SupplementaryFile1.docx]

**Supplementary Methods 1. Search Strategy and Evidence-Quality Assessment**

# **Search Date, Databases, and Time Range**

Searches covered database inception to June 2026. Databases and sources included PubMed/MEDLINE, Embase, PsycINFO, Web of Science Core Collection, the Cochrane Library, Google Scholar, prescribing information, clinical guidelines, and reference lists of key reviews.

**Full Boolean Search Strategies**

| **Source** | **Search string** | **Limits / notes** |
| --- | --- | --- |
| PubMed/MEDLINE | ("major depressive disorder"[Title/Abstract] OR depression[Title/Abstract] OR depressive symptoms[Title/Abstract]) AND (insomnia[Title/Abstract] OR "sleep disturbance"[Title/Abstract] OR "sleep maintenance"[Title/Abstract] OR "sleep latency"[Title/Abstract] OR "wake after sleep onset"[Title/Abstract] OR "early morning awakening"[Title/Abstract]) AND (hyperarousal[Title/Abstract] OR rumination[Title/Abstract] OR orexin[Title/Abstract] OR hypocretin[Title/Abstract] OR "sleep-wake regulation"[Title/Abstract]) AND (mirtazapine[Title/Abstract] OR trazodone[Title/Abstract] OR doxepin[Title/Abstract] OR "sedating antidepressant"[Title/Abstract] OR suvorexant[Title/Abstract] OR lemborexant[Title/Abstract] OR daridorexant[Title/Abstract] OR "dual orexin receptor antagonist"[Title/Abstract] OR DORA[Title/Abstract]) | Adults; English-language full text where available; no lower date limit. |
| Embase | ('major depressive disorder' OR depression OR 'depressive symptom') AND (insomnia OR 'sleep disorder' OR 'sleep disturbance' OR 'sleep maintenance' OR 'sleep latency' OR 'wake after sleep onset' OR 'early morning awakening') AND (hyperarousal OR rumination OR orexin OR hypocretin OR 'sleep wake regulation') AND (mirtazapine OR trazodone OR doxepin OR 'sedating antidepressant' OR suvorexant OR lemborexant OR daridorexant OR 'dual orexin receptor antagonist' OR DORA) | Human adults; articles, reviews, guidelines, and clinical trials. |
| PsycINFO | ("major depressive disorder" OR depression OR "depressive symptoms") AND (insomnia OR "sleep disturbance" OR "sleep maintenance" OR "sleep latency" OR "wake after sleep onset") AND (rumination OR hyperarousal OR orexin OR hypocretin) AND (mirtazapine OR trazodone OR doxepin OR suvorexant OR lemborexant OR daridorexant OR "dual orexin receptor antagonist") | Adult human studies; English-language records where available. |
| Web of Science Core Collection | TS=("major depressive disorder" OR depression OR "depressive symptoms") AND TS=(insomnia OR "sleep disturbance" OR "sleep maintenance" OR "sleep latency" OR "wake after sleep onset" OR "early morning awakening") AND TS=(hyperarousal OR rumination OR orexin OR hypocretin OR "sleep-wake regulation") AND TS=(mirtazapine OR trazodone OR doxepin OR suvorexant OR lemborexant OR daridorexant OR "dual orexin receptor antagonist" OR DORA) | All years to June 2026; article, review, and guideline records. |
| Cochrane Library | (depression OR "major depressive disorder") AND (insomnia OR "sleep disturbance") AND (mirtazapine OR trazodone OR doxepin OR suvorexant OR lemborexant OR daridorexant OR "orexin receptor antagonist") | Trials, reviews, and protocols relevant to insomnia, depression, or pharmacological treatment. |
| Google Scholar and reference lists | "major depressive disorder" insomnia DORA; depression insomnia mirtazapine trazodone doxepin; orexin insomnia depression; AASM insomnia doxepin trazodone guideline | First 200 Google Scholar records screened for relevance, supplemented by backward and forward citation tracking. |

# **Eligibility Criteria**

| **Criterion type** | **Definition** |
| --- | --- |
| Inclusion | Adult participants (>=18 years) with MDD, depressive symptoms, insomnia symptoms, insomnia disorder, or clinically relevant sleep disturbance. |
|  | Randomized trials, observational studies, systematic reviews, meta-analyses, clinical guidelines, prescribing labels, and mechanistic studies relevant to insomnia, hyperarousal, sedating antidepressants, DORAs, CBT-I, or safety. |
|  | Studies addressing at least one review domain: sleep phenotype, arousal mechanism, sedating antidepressant sleep effects, DORA efficacy/safety, dose intent, guideline positioning, or daytime function. |
| Exclusion | Animal studies, pediatric-only studies, case reports without broader mechanistic or safety relevance, conference abstracts without accessible full text, non-English articles when reliable extraction was not possible, and duplicate reports of the same dataset. |
|  | Primary neurological sleep disorders without depressive symptoms, healthy-volunteer pharmacology studies without clinical insomnia relevance, and articles focused exclusively on non-pharmacological interventions unless relevant as comparators or combined-care evidence. |

# **Screening Procedure**

Titles and abstracts were screened for relevance to MDD, insomnia symptoms or insomnia disorder, sleep-wake regulation, sedating antidepressants, DORAs, clinical guidelines, or safety. Full texts were reviewed when the abstract suggested relevance to drug selection, mechanism, dose, sleep outcomes, daytime function, or psychiatric safety. The final cited evidence map comprised 65 records.

| **Screening field** | **Number / status** | **Interpretation** |
| --- | --- | --- |
| Records in final cited evidence map | 65 | All references cited in the revised manuscript. |
| Records directly involving depression/MDD, depressive symptoms, or depression-related insomnia | Prioritized | Used to support statements about MDD where available. |
| General insomnia disorder RCTs, meta-analyses, guidelines, and prescribing labels | Included as indirect evidence | Used only when MDD-specific evidence was absent or insufficient. |
| Mechanistic or pharmacological studies | Included as hypothesis-generating evidence | Used to explain biological plausibility, not to validate treatment selection. |

# **Evidence-Quality Assessment Framework**

| **Evidence level** | **Evidence type** | **Use and limitation** |
| --- | --- | --- |
| Level 1: Direct clinical evidence | Studies in MDD, depressive symptoms, or depression-related insomnia with clinical sleep outcomes. | Most relevant for MDD positioning; still limited when phenotype moderators or head-to-head trials are absent. |
| Level 2: Indirect clinical evidence | General insomnia disorder RCTs, meta-analyses, or guideline recommendations. | Supports drug efficacy for insomnia outcomes but does not establish active-MDD treatment algorithms. |
| Level 3: Guideline or regulatory evidence | AASM guidelines and FDA prescribing information. | Used for treatment positioning, dose distinctions, contraindications, abuse potential, and suicide-risk warnings. |
| Level 4: Mechanistic or conceptual evidence | Studies on hyperarousal, sleep-wake regulation, orexin biology, rumination, or sleep architecture. | Supports biological plausibility only; cannot prove that symptoms identify mechanisms. |
| Level 5: Expert interpretation | Narrative synthesis and clinical reasoning based on convergent but indirect evidence. | Hypothesis-generating; should be framed as individualized clinical judgment rather than a validated algorithm. |
