# Supplementary material for: Pharmacological modulation of hyperarousal in insomnia associated with major depressive disorder: a narrative review of sedating antidepressants and orexin receptor antagonists
Source: Front Psychiatry. 2026 Sep 8;17:1928430. doi: 10.3389/fpsyt.2026.1928430 (PMC13595994; doi:10.3389/fpsyt.2026.1928430)
Supplement: Supplementary file 2 [file Table1.docx]

# Supplementary Table 1. Evidence map for pharmacological and comparator evidence relevant to MDD-related insomnia.

| **Drug/class** | **Citation(s) audited** | **Evidence type and population** | **Dose / treatment intent** | **Follow-up** | **Effect size / outcome direction** | **Risk of bias / key limitation** |
| --- | --- | --- | --- | --- | --- | --- |
| Sedating antidepressants as a class | Refs 18, 19, 21–24, 34, 35, 44 | Mixed evidence: polysomnographic meta-analysis, MDD/insomnia reviews, pharmacology reviews, and mood-disorder insomnia review evidence. Populations differ substantially and are not limited to active MDD. | Not a single dose category. Must separate antidepressant-dose treatment from low hypnotic-dose adjunctive use. | Variable across source types. | No single pooled effect size should be assigned to the class. Direction: sedating agents may improve subjective sleep or sleep continuity, but effects and harms vary by agent. | High clinical heterogeneity; indirectness; risk of overgeneralizing across mirtazapine, trazodone, doxepin, and TCAs. |
| Mirtazapine | Refs 19, 44, 46 | Direct/partly direct depression evidence, plus clinical pharmacology evidence. Relevant when depressive symptoms, appetite/weight loss, anxiety, and insomnia coexist. | Antidepressant-dose treatment; sleep effects partly related to H1 and 5-HT2 antagonism. Do not treat as low-dose hypnotic-only strategy. | Mostly acute to short-term antidepressant treatment periods; varies by cited study/review. | Direction: may improve subjective sleep quality and sleep continuity early in treatment. Exact standardized effect size not pooled in this narrative review because endpoints differ. | Sedation, weight gain, metabolic burden, and selection bias in clinical use; not phenotype-moderator evidence. |
| Trazodone | Refs 21, 24, 44, 45, 47 | Guideline plus heterogeneous insomnia/depression-adjacent evidence. Often used adjunctively for sleep, not as a primary antidepressant at hypnotic doses. | Low hypnotic doses commonly used for sleep should be separated from antidepressant-dose treatment. | Mostly short-term evidence in insomnia studies/guideline evidence base. | Direction: may improve subjective sleep continuity in some studies, but AASM guideline recommends against trazodone for chronic insomnia disorder. No MDD phenotype-specific effect size established. | Off-label use; heterogeneous trial quality; orthostasis, dizziness, and next-day impairment; low-dose use does not simultaneously treat depression. |
| Low-dose doxepin vs antidepressant-dose TCAs | Refs 18, 21, 24 | Guideline evidence for chronic insomnia disorder; broader depression/sleep evidence for antidepressant-dose TCAs. | Low-dose doxepin: 3 mg and 6 mg hypnotic intent, mainly H1-mediated. Antidepressant-dose doxepin/TCAs: broader receptor effects and higher safety burden. | AASM guideline evidence mainly from short-term insomnia trials; doxepin trials up to several weeks. | Direction: AASM supports doxepin for sleep-maintenance insomnia. Effect-size estimates should be read from the guideline/source trials; not pooled with antidepressant-dose TCAs. | Indirect for active MDD; risk if low-dose doxepin is grouped with antidepressant-dose TCAs or sleep-onset/global-hyperarousal pathway. |
| DORAs as a class | Refs 25–27, 48–50 | Mostly indirect evidence from insomnia disorder RCTs/meta-analyses and psychiatric-safety reviews; FDA labels provide safety/regulatory evidence. | Approved hypnotic doses differ by agent; not antidepressants and not mood-directed treatment. | Short-term to extension studies depending on agent; general-insomnia evidence dominates. | Direction: consistent improvement in WASO, total sleep time, and sleep efficiency in insomnia disorder. MDD-specific effect size and phenotype-moderator effect not established. | Indirectness for active MDD; unstable psychiatric illness/high suicide risk often underrepresented; controlled-substance and label safety concerns. |
| Suvorexant | Refs 25, 50–52, 58 | Indirect insomnia-disorder RCT/long-term evidence plus FDA prescribing information; active MDD not the primary trial population. | Approved hypnotic dosing; Schedule IV controlled substance. Typical approved adult maximum is 20 mg; older pivotal trials also evaluated higher doses. | Three-month pivotal trial evidence and longer-term safety data in insomnia disorder. | Direction: improves sleep onset/maintenance outcomes in insomnia disorder, especially subjective total sleep time and wakefulness after sleep onset. No active-MDD phenotype effect size established. | Psychiatric generalizability limited; label warning for worsening depression/suicidal ideation and abuse potential. |
| Lemborexant | Refs 25, 27, 50, 53, 54, 56, 59 | Indirect insomnia-disorder RCT evidence, sleep-architecture evidence, subgroup evidence involving depression/anxiety medications, and FDA prescribing information. | 5 mg or 10 mg hypnotic dosing; Schedule IV controlled substance. | Short-term and longer-term insomnia trial data depending on study; subgroup evidence remains limited. | Direction: improves sleep onset and maintenance outcomes and has sleep-architecture data. Effect in active MDD and suicidality outcomes not established. | Psychiatric subgroup not equivalent to active-MDD trial; label warns about worsening depression/suicidal ideation and abuse potential. |
| Daridorexant | Refs 25–27, 50, 55, 57 | Indirect general-insomnia RCT/post hoc/extension evidence; systematic review context for mood-disorder relevance. | Approved hypnotic dosing; phase 3 evidence includes 25 mg and 50 mg, with 50 mg most consistently linked to daytime-function outcomes. | Twelve-week phase 3 trials; extension studies report longer-term safety/tolerability. | Direction: improves sleep maintenance/sleep latency; 50 mg also improves daytime functioning in insomnia disorder. Active-MDD effect size not established. | Indirectness for MDD; post hoc architecture/function outcomes may not translate to depression relapse or suicidality. |
| CBT-I and combined care | Refs 14, 15, 20, 22, 23, 60–62, 64 | Direct depression/insomnia evidence plus insomnia-disorder guideline evidence; includes behavioral and combined-care sources. | Non-pharmacological intervention; session number and format vary. Combination with medication depends on clinical context. | Short-term treatment trials and longer-term follow-up vary by source. | Direction: generally improves insomnia severity and may enhance depression outcomes in comorbid MDD/insomnia. Effect sizes vary by outcome and meta-analysis. | Intervention heterogeneity; sequencing with DORAs or sedating antidepressants remains uncertain. |
| Clinical framework / phenotype-based selection | Refs 18, 19, 21, 23, 25–27, 31, 44, 48, 52, 57, 61, 62 | Synthesis of direct, indirect, guideline/regulatory, and mechanistic evidence. | Not dose-specific; applies to clinical assessment and shared decision-making. | Not applicable. | No moderator studies or head-to-head active-MDD trials validate phenotype-to-drug assignment. | Main risk is overinterpretation: symptoms may suggest hypotheses but do not identify mechanisms. |

*Abbreviations: CBT-I, cognitive behavioral therapy for insomnia; DORAs, dual orexin receptor antagonists; MDD, major depressive disorder; REM, rapid eye movement; TCAs, tricyclic antidepressants; WASO, wake after sleep onset.*
